# The Physiological Mechanisms of Performance Enhancement with Sprint Interval Training Differ between the Upper and Lower Extremities in Humans

**DOI:** 10.3389/fphys.2016.00426

**Published:** 2016-09-30

**Authors:** Christoph Zinner, David Morales-Alamo, Niels Ørtenblad, Filip J. Larsen, Tomas A. Schiffer, Sarah J. Willis, Miriam Gelabert-Rebato, Mario Perez-Valera, Robert Boushel, Jose A. L. Calbet, Hans-Christer Holmberg

**Affiliations:** ^1^Department of Sport Science, Julius Maximilians University WürzburgWürzburg, Germany; ^2^Swedish Winter Sports Research Centre, Mid Sweden UniversityÖstersund, Sweden; ^3^Research Institute of Biomedical and Health Sciences (IUIBS) and Department of Physical Education, University of Las Palmas de Gran CanariaLas Palmas, Spain; ^4^Institute of Sports Science and Clinical Biomechanics, University of Southern DenmarkOdense, Denmark; ^5^Swedish School of Sport and Health SciencesStockholm, Sweden; ^6^Department of Medical and Health Sciences, Linköping UniversityLinköping, Sweden; ^7^School of Kinesiology, University of British ColumbiaVancouver, BC, Canada; ^8^School of Sport Sciences, UiT Arctic University of NorwayTromsø, Norway

**Keywords:** high-intensity training, lower body, performance, triceps brachii, upper body

## Abstract

To elucidate the mechanisms underlying the differences in adaptation of arm and leg muscles to sprint training, over a period of 11 days 16 untrained men performed six sessions of 4–6 × 30-s all-out sprints (SIT) with the legs and arms, separately, with a 1-h interval of recovery. Limb-specific VO_2_peak, sprint performance (two 30-s Wingate tests with 4-min recovery), muscle efficiency and time-trial performance (TT, 5-min all-out) were assessed and biopsies from the *m. vastus lateralis* and *m. triceps brachii* taken before and after training. VO_2_peak and Wmax increased 3–11% after training, with a more pronounced change in the arms (*P* < 0.05). Gross efficiency improved for the arms (+8.8%, *P* < 0.05), but not the legs (−0.6%). Wingate peak and mean power outputs improved similarly for the arms and legs, as did TT performance. After training, VO_2_ during the two Wingate tests was increased by 52 and 6% for the arms and legs, respectively (*P* < 0.001). In the case of the arms, VO_2_ was higher during the first than second Wingate test (64 vs. 44%, *P* < 0.05). During the TT, relative exercise intensity, HR, VO_2_, VCO_2_, V_E_, and V_t_ were all lower during arm-cranking than leg-pedaling, and oxidation of fat was minimal, remaining so after training. Despite the higher relative intensity, fat oxidation was 70% greater during leg-pedaling (*P* = 0.017). The aerobic energy contribution in the legs was larger than for the arms during the Wingate tests, although VO_2_ for the arms was enhanced more by training, reducing the O_2_ deficit after SIT. The levels of muscle glycogen, as well as the myosin heavy chain composition were unchanged in both cases, while the activities of 3-hydroxyacyl-CoA-dehydrogenase and citrate synthase were elevated only in the legs and capillarization enhanced in both limbs. Multiple regression analysis demonstrated that the variables that predict TT performance differ for the arms and legs. The primary mechanism of adaptation to SIT by both the arms and legs is enhancement of aerobic energy production. However, with their higher proportion of fast muscle fibers, the arms exhibit greater plasticity.

## Introduction

High-intensity training (HIT), and in particular sprint-interval training (SIT) with the legs, effectively improves the performance of untrained individuals and recreational athletes (Edge et al., 2006; Gibala et al., 2006; Amundsen et al., 2008; Jacobs et al., 2013), as well as of elite athletes (Stepto et al., 1999; Laursen et al., 2005; Lamberts et al., 2009). For example, following six SIT sessions over a 2-week period, untrained individuals exhibited better performance (Burgomaster et al., 2005, 2006; Gibala et al., 2006; Little et al., 2010), associated with elevated activities and levels of mitochondrial enzymes, as well as enhanced levels of resting glycogen, and glucose and fatty acid transport proteins in their muscles (Burgomaster et al., 2005; Gibala et al., 2006; Vincent et al., 2015) and more extensive capillarization (Cocks et al., 2013). Analysis of myosin heavy chains (MHC) revealed a transformation from glycolytic (IIx) to glycolytic-oxidative (IIa) muscle fibers after only 15 bouts of leg sprint exercise over a 6-week period (Allemeier et al., 1994). However, it remains unknown whether the arms respond to SIT in a similar manner.

The forms of exercise most commonly used in SIT studies involve the legs, e.g., cycling and running [for a review, see Gist et al., 2014] and only one such investigation has focused on whole-body exercise (rowing) (Carr, 2011) and none on work by the arms alone. In most people, the upper body has less muscle mass and performs less work than the legs, resulting in differences in metabolic and cardiovascular responses (Miles et al., 1989; Calbet et al., 2015a). These differences include more pronounced heterogeneity in blood flow, a shorter mean transit time for the blood, a smaller diffusion area with larger diffusion distances (Calbet et al., 2005; Stöggl et al., 2013), and less vascular reactivity in the upper body during exercise (Richardson et al., 2006), as well as a lower cardiac output, but more pronounced cardiovascular strain during upper- than lower-body exercise (Calbet et al., 2015a).

Most muscle groups in the upper body contain a greater proportion of fast-twitch fibers than those in the lower body (Koppo et al., 2002; Sanchís-Moysi et al., 2010) and, thus, adaptation by the arms and legs to SIT may differ. It remains to be determined whether upper-body muscles, with their greater proportion of type II fibers, rely more heavily on anaerobic energy than the leg muscles during sprint exercise. Clearly, endurance cyclists rely more heavily on oxidative metabolism than sprint cyclists (Calbet et al., 2003). Indeed, during isolated sprint tests total oxygen consumption (VO_2_) by the upper body is greater than that by the lower body, when adjusted for power output (Price et al., 2014). However, recent measurements using the direct Fick procedure demonstrated similar VO_2_peak values per kg of muscle for the arms and legs of trained individuals during arm-cranking and leg-pedaling (Calbet et al., 2015a). Thus, the factors limiting performance during upper- and lower-body exercise differ somewhat and adaptations to training can also be expected to be slightly different.

Although HIT and, in particular, SIT is commonly employed by elite athletes to enhance performance, both of these training modalities are now being recommended in some cases for recreational athletes, as well as sedentary individuals, and patients with chronic diseases (Wahl et al., 2010). In this context when training with the legs is not possible, or when it is an aim to include upper body training for health or performance improvements it is of interest whether training with the arms/upper body might improve endurance within a short period of time as well. Since the metabolic and health benefits of training are dependent on the amount of muscle mass employed, training both the upper- and lower-body may be beneficial, especially for untrained individuals and those with relatively little muscle mass in their lower body. Furthermore, in connection with a variety of sports considerable work is generated by the upper body (e.g., kayaking, cross-country skiing, swimming) and little is known about the effects of SIT employing the upper body alone or in combination with the lower body.

Therefore, the current investigation was designed to determine whether the arms and legs adapt differently to the same short-term SIT. A secondary aim was to compare the key determinants of the performance of high-intensity endurance exercise by the arms or legs only. Our hypothesis was that anaerobic capacity would be enhanced in both sets of limbs, but to a greater extent in the arms, whereas the improvement in oxidative capacity would be more pronounced in the legs.

## Materials and methods

### Subjects

The 16 healthy male participants (24 ± 4 yrs; 184 ± 7 cm; 80 ± 14 kg) all exercised recreationally (jogging, cycling, etc.) two or three times each week, but none trained regularly for any particular sporting event or performed exercise involving primarily the upper body (Table 1). They were all fully informed of the nature of the study, which was pre-approved by the Regional Ethical Review Board in Umeå, Sweden (2014/91-31), before providing their written consent to participate.

**Table 1:** **Characteristics of the participants (means ± SD) before (pre) and after (post) the training intervention**.

	**Pre**	**Post**
Age (yrs)	24 ± 4	
Body mass (kg)	77.8 ± 6.9	77.7 ± 6.8
Total body lean mass (kg)	60.9 ± 5.8	61.1 ± 5.8
Percentage of fat mass for the entire body (%)	18.1 ± 4.9	17.8 ± 4.7
VO_2_peak (leg exercise) (L·min^−1^)	3.9 ± 0.5	4.1 ± 0.5^*^
VO_2_peak (leg exercise) (mL·kg^−1^·min^−1^)	48.7 ± 6.5	51.9 ± 7.2^*^

* *P < 0.05 compared to the Pre-value*.

### Experimental protocol

**Figure 1:**
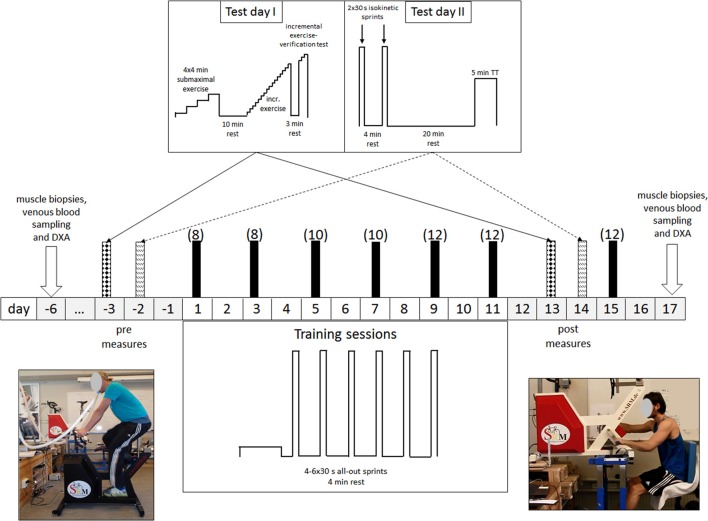
**Schematic illustration of our experimental protocol**. All testing and training was performed employing both leg-pedaling (left) and arm-cranking (right). The values in brackets indicate the number of 30-s sprints per training session.

As illustrated in Figure 1, our experimental protocol involved, in chronological order, (1) taking muscle biopsies and venous blood samples (pre-biopsies); (2) measurements of performance before the intervention (pre-testing); (3) an 11-day period of training; (4) subsequent performance measurements (post-testing); (5) one more session of training; and (6) at least 48 h later, collecting new muscle biopsies and venous blood samples (post-biopsies). Body composition was assessed on the same days as the pre- and post-biopsies were taken. The testing (pre and post) included limb-specific incremental tests for determination of VO_2_peak, two 30-s Wingate tests separated by a 4-min recovery, a 4 × 4-min submaximal incremental exercise test (from 40 to 80–90% of VO_2_ max with one bout at 80 W) designed to determine work efficiency, and a 5-min all-out time trial (TT).

Before the experiment itself, each subject visited the laboratory twice to become familiarized with the procedures and training ergometers. In connection with these visits, individual settings for the leg cycling ergometer (Schoberer Rad Meßtechnik SRM GmbH, Jülich, Germany) and an ergometer modified for arm cycling (Figure 1; Schoberer Rad Meßtechnik SRM GmbH, Jülich, Germany) were determined and used in all subsequent testing and training. For arm cycling the saddle was arranged so that the scapula-humeral joint and the axle of the crank were at the same level and the elbow angle comfortable when the cranks were in a horizontal position (Brink-Elfegoun et al., 2007).

### Assessment of body composition

Body composition was assessed using dual-energy x-ray absorptiometry (Lunar iDXA, GE Healthcare, Madison, WI, USA), calibrated in accordance with the manufacturer's guidelines. Limb lean mass (kg), calculated from regional analysis of the whole-body scan, served as a surrogate measure of the muscle mass of the arms and legs, as reported previously (Calbet et al., 2015c). In brief, from the whole-body DXA-scan, the region of the upper limbs was delineated by a line bisecting the glenohumeral joint and the acromioclavicular joints. The lower extremities were delineated by a line crossing the neck of the femur below the iliac bone as close as possible to the anterosuperior iliac spine and ischium.

### Pre- and post-training tests

The subjects reported to the laboratory after an overnight fast and then, in randomized order, half first performed all of the tests with the arms and 2 h later repeated the tests with the legs, while the other half began with the legs. On the first day of testing (Day 1), each participant performed a submaximal incremental test consisting of four 4-min bouts at 100 rpm, with individual intensities such that the highest evoked a respiratory exchange ratio (RER) close to 1.0. The same absolute intensities were utilized in pre- and post-testing.

Ten minutes after this submaximal incremental test, each participant performed an incremental test to exhaustion to determine VO_2_peak. In the case of the arms, this test started at 20 W and the intensity was increased by 10 W each 30 s; while for the legs, the initial intensity was 60 W and the increase 25 W every 30 s. The criteria for VO_2_peak for the arms and legs were (1) a plateau in oxygen uptake (i.e., an elevation of less than 2.1 mL·min^−1^·kg^−1^ with increasing resistance), (2) a RER > 1.10, (3) a heart rate within 2.5% of the age-adjusted maximal, and (4) a capillary blood lactate concentration after the exercise > 6 mmol·L^−1^. In all cases, at least 3 of these 4 criteria were fulfilled.

After experiencing exhaustion, the subject remained seated on the ergometer, slowly moving the limbs that had just been exercised. Three minutes later, the intensity was increased to the highest level attained during the incremental exercise +10 W for the arms and +25 W for the legs, to confirm that VO_2_peak had actually been achieved. During both the incremental exercise to exhaustion and the subsequent verification tests, the subjects were allowed to choose their own comfortable rate of pedaling/arm cranking within the range of 70–105 rpm. No feedback concerning performance, other than pedaling rate, was provided during either the pre- or post-test, but the subjects were encouraged strongly to resist fatigue and exert themselves as much as possible. Exhaustion was defined as the inability to maintain a rate faster than 70 rpm for 5 s, despite strong verbal encouragement.

During the second day of testing (Day 2) the volunteers performed two 30-s all-out isokinetic sprints at 100 rpm (Wingate anaerobic tests; WAnT), separated by 4 min of recovery. They then rested for 20 min to allow full recovery of muscle lactate and pH (Bangsbo et al., 1993), and thereafter performed a 5-min time-trial (TT) at 100 rpm. In randomized order, half of the subjects performed the leg WAnTs (+leg TT) first and, after 2 h of rest, the arm WAnTs (+arm TT), while the other half started with the arms. Strong verbal encouragement was provided to subjects during the WAnTs and the TTs. During the WAnTs the subjects were not allowed to stand up. From the WAnTs, the peak (PPO) and mean power outputs (MPO) were determined and the fatigue index (FI, decline in power) calculated as FI = [(Peak Power - Lowest Power) 100]·Peak Power^−1^. The MPO developed during the TT was employed as an index of endurance performance (Currell and Jeukendrup, 2008).

### Cardiorespiratory variables

During the incremental test, as well as both WAnTs and the TT, the oxygen uptake (VO_2_), carbon dioxide production (VCO_2_), and pulmonary ventilation (V_*E*_) were monitored continuously and averaged every 10 s employing an open-circuit metabolic cart (AMIS 2001 model C, Innovision A/S, Odense, Denmark) calibrated prior to each test with a 3-liter syringe (Hans Rudolph Inc., Kansas City, KS, USA) and a certified mixture of 16.00% O_2_ and 4.50% CO_2_ in N_2_. Heart rate (HR) was also monitored (RS400, Polar Electro Oy, Kempele, Finland) during all testing. Before and immediately, as well as 1.5 and 3 min after both WAnTs, and immediately after the TT, capillary blood samples were taken from the earlobe for determination of the lactate concentration (BLa) utilizing an automated system (Biosen 5140, EKF Diagnostic GmbH, Magdeburg, Germany). VO_2_peak was defined as the highest average 20-s VO_2_ value during either the incremental exercise, the verification test or the TT.

### Calculation of the efficiencies of pedaling and arm-cranking, oxidation of fat, and oxygen deficit

The O_2_ demand was calculated from the linear relationship between VO_2_ and exercise intensity observed during the submaximal incremental exercise [the average of the last 2 min; from 40 to 80–90% of VO_2_max (for RER < 1)]. The gross efficiency was calculated as the work performed/total energy expenditure. The delta efficiency was calculated as the ratio of the change in work accomplished per minute and the change in energy expended per minute. Gross and delta efficiencies pre- and post-training were determined using the same absolute loads for each subject. Oxidation of fat was determined by indirect calorimetry (Massicotte et al., 1992). The oxygen deficit (OD), i.e., the difference between O_2_ demand and VO_2_ during the Wingate test, was determined as reported previously (Calbet et al., 1997a; Dorado et al., 2004).

### Training protocol

Each of the 6 days of training, all conducted within an 11-day period (Figure 1), involved one session of arm and one session of leg cycling, separated by 1 h of recovery. Half of the subjects trained first with the arms while the other half started first with the legs. For both the arms and legs, the subject was instructed to perform (4–6) repeated 30-s all-out sprints (a Wingate anaerobic test), separated by 4 min of recovery (at unloaded pedaling or cranking at ~20 rpm). On the first and second days of training, each subject performed four sprints; on the third and fourth days, five sprints; and on the final 2 days, as well as during the extra training session, six sprints (Figure 1), for a total of 36 sprints each of arm and leg cycling.

All training sessions were supervised by one of the researchers. After every training session (arm or leg), ratings of perceived exertion (RPE) were obtained for the whole body and the limbs trained. Before and after the first, third, and fifth days of training a blood sample was taken for determination of lactate.

### Collection and preparation of the muscle biopsies

Before and after the training intervention (see Figure 1), muscle biopsies were taken in randomized order from the m. *vastus lateralis* and m. *triceps brachii* on the left side of the body of half of the subjects and the right side for the other half. All biopsies were collected by the same person to ensure a standard localization and muscle depth. The subjects reported to the laboratory at the same time of day following an overnight fast; local anesthesia (2–3 mL 2% carbocaine) was applied; and a biopsy taken through an incision in the skin and fascia of the m. *vastus lateralis* and m. *triceps brachii* (distal portion of the lateral head), employing a modified Bergström needle with suction. These muscles were selected because they are intensely active during arm and leg cycling, respectively (Lusina et al., 2008; Torres-Peralta et al., 2014).

The muscle tissue thus obtained was dried on filter paper; placed on a glass plate cooled on ice; and freed of visible blood, connective tissue, and fat. Approximately half of the tissue was frozen immediately in liquid nitrogen and stored at −80°C for later analysis. The other half was divided into five pieces, two of which were immediately frozen in liquid N_2_ and stored at −80°C for later analyses of glycogen and enzyme activities. The third piece was weighed and homogenized in 10 volumes (w/v) of ice-cold buffer (300 mM sucrose, 1 mM EDTA, 10 mM NaN_3_, 40 mM Tris-base, and 40 mM histidine at pH 7.8) in a 1-mL glass homogenizer with a glass pestle (Kontes Glass Co., Vineland, NJ, USA). This homogenate was then divided into aliquots that were frozen in liquid nitrogen and stored at −80°C for later analysis of the heavy MHC composition. A fourth piece was mounted on cork blocks with Tissue-Tek O.C.T.™ embedding medium and oriented so that myofibers could be cut transversely. Specimens were frozen by immersion (10 s) in isopentane, followed by storage in liquid nitrogen. Finally, a small (30–40 mg) fragment was prepared for assessment of mitochondrial function *in vitro* using high-resolution respirometry, as described in a separate article (Larsen et al., 2016).

### Analytical procedures

#### Enzyme activities

Citrate synthase (CS) and 3-hydroxyacyl-CoA dehydrogenase (HAD) were assayed at 25°C (Lowry and Passonneau, 1972). CS activity was determined in the presence of oxaloacetate, acetyl-CoA, and DTNB buffer; and HAD activity with acetoacetyl-CoA and NADH in a buffer solution containing imidazole and EDTA. In both cases, the change in absorbance at 340 nm was recorded for 600 s, converted into enzyme activity and expressed as micromoles per gram of dry weight per minute.

#### The glycogen content of muscle

For spectrophotometric determination of glycogen (Beckman DU 650), freeze-dried muscle tissue (approximately 1.5 mg) was boiled in 0.5 mL 1 M HCl for 150 min, quickly cooled, vortexed, and centrifuged (3500 g, 9.5 min, 4°C). Forty microliters of the supernatant thus obtained and 1 mL reagent solution containing Tris buffer (1 M), ATP (100 mM), MgCl_2_ (1 M), NADP^+^ (100 mM), and glucose-C-phosphate dehydrogenase were mixed before initiating the assay with 10 μL diluted hexokinase. The change in absorbance at 340 nm was recorded for 60 min and the glycogen content calculated as mmoles per kilogram of dry weight.

#### Fiber type distribution

The MHC composition of the homogenate was determined using gel electrophoresis (Ortenblad et al., 2000). Muscle homogenate (80 μL) was mixed with 200 μL of sample buffer 10% glycerol, 5% 2-mercaptoethanol, and 2.3% sodium dodecyl sulfate (SDS), 62.5 mM Tris, and 0.2% bromophenol blue (at pH 6.8), boiled in water for 3 min, and loaded (10–40 μL) onto an SDS–PAGE gel [6% polyacrylamide (100:1 acrylamide–bis-acrylamide), 30% glycerol, 67.5 mM Tris base, 0.4% SDS, and 0.1 M glycine]. Gels were run at 80 V for at least 42 h at 4°C, and MHC bands visualized by staining with Coomassie. The gels were scanned (Lino-scan 1400 scanner, Heidelberg, Germany) and MHC bands quantified densitometrically (Phoretix 1D, non-linear, Newcastle, UK) as the average from the three amounts of protein loaded. MHC-II was identified by Western blotting using monoclonal antibody (Sigma M4276) in the protocol Xcell IITM (Invitrogen, Carlsbad, CA, USA).

#### Histochemical analysis of fiber type and size and capillarization

Serial sections (8 μm) of the muscle samples (mounted in Tissue-Tek O.C.T.™) were cut in a cryostat (−20°C) and carefully placed onto microscope slides. To determine the fiber composition, adenosine triphosphate (ATPase) was analyzed histochemically after pre-incubation at pH values of 4.37, 4.60, and 10.30 (Brooke and Kaiser, 1970). To assess capillary density, another slide was stained employing the amylase-para-aminosalicylic acid procedure, which was optimized by adding hematoxylin at the end (Andersen, 1975). Pre- and post-slides from each limb from the same subject were incubated at the same time to reduce variability.

Only fibers that had actually been cut horizontally were employed for determination of fiber size. A mean of 228 ± 79 fibers in each biopsy were examined. In 2–4 individuals (depending on the extremity and training status) the number of IIx fibers was too low to allow reliable statistical analysis. The serial sections were visualized and analyzed with an Olympus BX40 microscope and Olympus camera (DP 26) (Olympus Optical Co., Tokyo, Japan), with image analysis software (Olympus CellSens Standard, Tokyo, Japan).

Using the ATPase staining at pH 4.60, a fiber pattern was drawn manually by following the boundaries of the fibers and this pattern then superimposed on the other stainings. Fiber types were identified on the basis of their staining properties at the different pHs and the cross-sectional area (CSA) determined. The relative area of each fiber type was calculated as the product of average size times percentage distribution. The capillaries per unit area of the amylase-PAS sections were counted.

### Statistical analyses

All variables were shown to exhibit a normal distribution using the Shapiro Wilk's test. Descriptive values are presented as means ± standard deviations (SD). The differences between pre- and post-training and between the arms and legs were compared using repeated-measures ANOVA. Pair-wise comparisons at specific time-points were adjusted for multiple comparisons with the Holm-Bonferroni procedure. The relative changes in VO_2_peak in the arms and legs were compared using a *t*-test. Associations between variables were evaluated using Pearson's correlation coefficient and the variables that predicted time-trial performance identified by stepwise multiple linear regression analysis. All of these analyses were performed in the SPSS v.17.0 software for Windows (SPSS Inc., Chicago, IL, USA).

## Results

### Cardiovascular variables associated with exercise by the arms or legs

Although maximal heart rate was similar during the incremental exercise by the arms and legs, Wmax, and VO_2_peak were 84 and 21% higher, respectively, in the legs. Conversely, in relationship to lean muscle mass, the corresponding values were 38 and 209% higher for the arms. The maximal ventilatory response to leg exercise was 24% greater than for the arms, but proportional to the VO_2_ and VCO_2_, as indicated by the similar V_*E*_/VO_2_ and V_E_/VCO_2_ ratios for both sets of limbs at Wmax. The higher V_E_ during leg exercise could be explained by a 28% greater tidal volume, whereas the respiratory rate (RR) was similar in both cases.

### Training load and lactate responses

**Figure 2:**
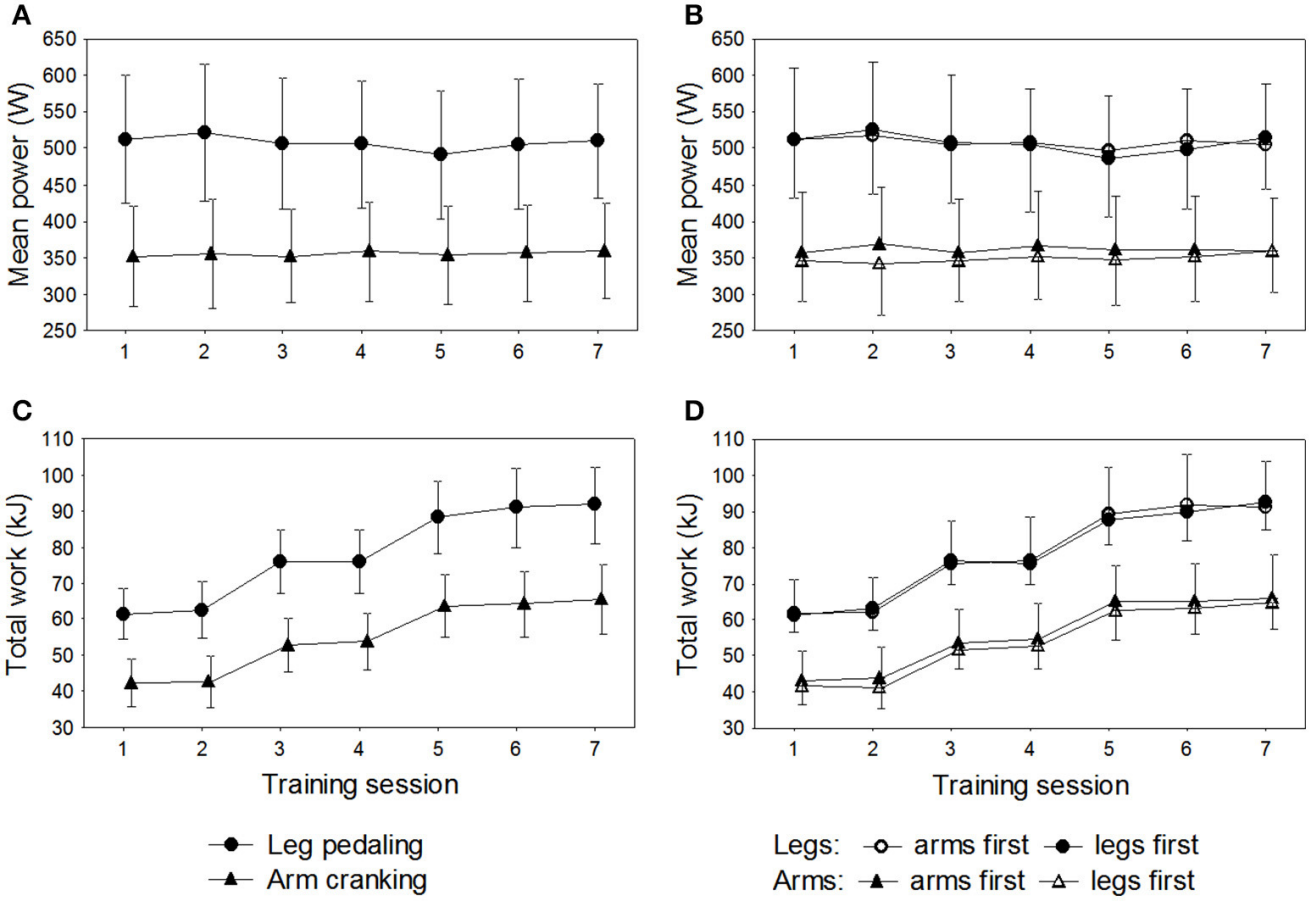
**Results for the 16 subjects during the seven training sessions of leg-pedaling and arm-cranking**. **(A)** Mean power output, **(B)** mean power output in relationship to order of training, **(C)** total work, and **(D)** total work in relationship to order of training.

All 16 participants completed the 72 all-out sprints (36 with arm cycling and 36 with leg cycling) during the 7 training sessions. The overall mean power output was 356 ± 67 W for arm cycling and 507 ± 87 W for leg cycling (*P* < 0.001) and the corresponding peak power outputs 547 ± 102 W and 767 ± 129 W (*P* < 0.001). The peak levels of blood lactate after training sessions 1, 3, and 5 were significantly higher with the legs than arms (overall mean lactate levels after the training sessions: 14.8 ± 2.4 mmol·L^−1^ vs. 12.6 ± 2.3 mmol·L^−1^, *P* < 0.001). The ratings of perceived whole-body exertion (RPE) were 16.2 ± 1.5 for the arm and 17.0 ± 1.8 for leg cycling, with corresponding ratings of 18.3 ± 1.3 and 18.8 ± 1.2 for exertion by the arms and legs, respectively. The total amount of work performed during the sessions of leg training was 546 ± 43.5 and 547 ± 80.8 kJ for the groups training first with the legs or arms, respectively (*P* = 0.94). The total work performed during the sessions of arm training was 377 ± 44.3 and 392 ± 66.2 kJ for the groups training first with the legs or arms, respectively, with no difference between the two sets of limbs (*P* = 0.61) (Figure 2).

### Effects of training

#### Body composition (Table 1)

The training intervention had no effect on weight, lean mass or % fat of the whole body, arms, or legs.

#### The incremental test

Performance and ergospirometric variables during the incremental exercise test are summarized in Table 2. After training, VO_2_peak was enhanced by 9.8 and 6.1% for the arms and legs, respectively (*P* = 0.03). Similar improvements were observed in Wmax, VO_2_peak·Wmax^−1^, and VO_2_peak·limb lean mass^−1^ in association with both modes of exercise. However, the improvements in Wmax, exercising time, and VO_2_peak·limb lean mass^−1^ were more pronounced for the arms (Training × Extremity, *P* < 0.05). Training had no significant effects on peak heart rate.

**Table 2:** **Effects of six sessions of sprint interval training on ergospirometric variables and performance during incremental exercise to exhaustion, exercise at 80 W, and efficiency**.

**Variable**	**Arms**		**Legs**		**Main training effect**	**Main extremity effect**	**Training × Extremity interaction**
	**Pre-training**	**Post-training**	**Pre-training**	**Post-training**			
**INCREMENTAL EXERCISE TEST**							
Wmax (W)	187.4 ± 28.8	207.1 ± 32.2^a^	356.6 ± 40.5	370.1 ± 42.2^a^	0.000	0.000	0.019
Wmax·limb mean mass^−1^ (W·kg^−1^)	22.91 ± 2.90	25.31 ± 2.60^a^	17.21 ± 1.41	17.81 ± 1.53^a^	0.000	0.000	0.000
T lim (s)	502.1 ± 86.5	561.3 ± 96.6	355.9 ± 48.6	372.1 ± 50.6	0.000	0.000	0.000
HR (beats·min^−1^)	186.0 ± 10.1	187.8 ± 8.4	187.4 ± 9.3	188.8 ± 7.9	0.272	0.209	0.801
VO_2_peak (L·min^−1^)	3.15 ± 0.48	3.46 ± 0.48^a^	3.88 ± 0.47	4.12 ± 0.46^a^	0.000	0.000	0.184
VO_2_peak·Wmax^−1^ (mL·min^−1^·W^−1^)	16.8 ± 1.2	16.8 ± 1.2	39.6 ± 6.1	40.2 ± 3.9	0.705	0.000	0.645
VO_2_peak·lean mass^−1^ (mL·min^−1^·kg^−1^)	383.8 ± 44.8	422.4 ± 36.5^a^	186.8 ± 12.8	198.0 ± 15.7^a^	0.000	0.000	0.000
VCO_2_ (L·min^−1^)	3.50 ± 0.55	3.91 ± 0.59^a^	4.57 ± 0.65	5.08 ± 0.85^a^	0.000	0.000	0.401
V*_E_* (L·min^−1^)	121.5 ± 19.6	136.1 ± 20.5^a^	153.4 ± 30.5	165.0 ± 21.1	0.001	0.000	0.653
RER	1.15 ± 0.06	1.16 ± 0.04	1.19 ± 0.11	1.25 ± 0.11	0.163	0.028	0.161
RR (breaths·min^−1^)	55.7 ± 8.4	58.9 ± 8.2	54.4 ± 12.1	58.2 ± 5.7	0.055	0.626	0.825
Vt (L)	2.44 ± 0.41	2.46 ± 0.37	3.18 ± 0.56	3.08 ± 0.42	0.507	0.000	0.137
V*_E_*/VO_2_	38.8 ± 4.8	39.7 ± 5.2	39.6 ± 6.1	40.2 ± 3.9	0.371	0.532	0.840
V*_E_*/VCO_2_	34.8 ± 3.9	35.1 ± 5.0	34.0 ± 6.8	33.0 ± 5.0	0.727	0.173	0.400
Blood lactate 2.5 min (mM)	9.99 ± 2.05	10.95 ± 1.71^a^	10.39 ± 2.43	10.76 ± 2.04	0.049	0.771	0.147
**EXERCISE AT 80 W**							
HR (beats·min^−1^)	152.20 ± 21.40	135.60 ± 17.06^a^	132.13 ± 17.72	129.73 ± 14.71	0.000	0.011	0.001
VO_2_ (L·min^−1^)	1.94 ± 0.22	1.75 ± 0.15^a^	2.06 ± 0.33	2.04 ± 0.30	0.008	0.014	0.010
VO_2_·lean mass^−1^ (mL·min^−1^·kg^−1^)	238.78 ± 29.08	216.38 ± 26.06^a^	99.12 ± 12.46	98.12 ± 11.63	0.009	0.000	0.007
VCO_2_ (L·min^−1^)	1.95 ± 0.18	1.73 ± 0.14^a^	1.94 ± 0.41	1.90 ± 0.30	0.002	0.355	0.033
V*_E_* (L·min^−1^)	57.99 ± 4.81	51.10 ± 5.78^a^	51.11 ± 13.26	50.37 ± 9.53	0.010	0.138	0.015
RER	1.01 ± 0.05	0.99 ± 0.04	0.94 ± 0.07	0.93 ± 0.05	0.212	0.001	0.668
RR (breaths·min^−1^)	28.9 ± 4.2	28.4 ± 5.6	24.4 ± 5.1	24.8 ± 4.3	0.861	0.000	0.460
V*_t_* (L)	2.04 ± 0.29	1.85 ± 0.35^a^	2.13 ± 0.47	2.06 ± 0.32	0.001	0.019	0.309
V*_E_*/VO_2_	30.0 ± 3.2	29.3 ± 3.6	24.7 ± 3.4	24.7 ± 2.6	0.397	0.000	0.128
V*_E_*/VCO_2_	29.8 ± 2.0	29.5 ± 3.0	26.2 ± 2.5	26.6 ± 2.6	0.953	0.000	0.226
Blood lactate (mM)	4.84 ± 1.32	4.45 ± 1.36	2.48 ± 1.29	2.26 ± 0.72	0.101	0.000	0.652
**EFFICIENCY**							
Intensity (W)	67.5 ± 11.1	67.5 ± 11.1	120.3 ± 24.4	120.3 ± 24.4			
Gross efficiency	0.11 ± 0.01	0.11 ± 0.01	0.14 ± 0.02	0.13 ± 0.02	0.004	0.000	0.000
Delta efficiency	0.17 ± 0.02	0.17 ± 0.02	0.24 ± 0.04	0.24 ± 0.05	0.426	0.000	0.716

Values are means ± SD; n = 16 subjects; Wmax (W), Peak power output during the incremental exercise test; T lim, duration of the incremental exercise test (s); HR, Heart rate; VO_2_peak, Peak oxygen uptake during the incremental exercise test; VCO_2_, CO_2_ production; V_E_, Pulmonary ventilation; RER, Respiratory exchange ratio; RR, Respiratory rate; V_t_, Tidal volume;

a *P < 0.05 compared corresponding Pre-training value*.

The improvement in VO_2_peak was associated with a greater V_*E*_ due to a higher RR, with similar responses of tidal volume (V_t_) to maximal exercise before and after training. This rise in V_E_ was proportional to the increases in VO_2_ and VCO_2_, as reflected in the similar V_E_/VO_2_ and V_E_/VCO_2_ ratios before and after training. The peak level of lactate in the blood, measured 2.5 min after exhaustion, was 9.6% higher after arm-cranking, but unchanged after cycling (Table 2) compared to pre-training values.

#### Constant exercise at 80 W

Metabolic variables were determined at 80 W, i.e., 62 and 53% of VO_2_peak for the arms and legs, respectively, before training; and 51 and 50% of VO_2_peak for the arms and legs respectively, after training. Prior to the training intervention, the O_2_ cost of exercise at 80 W was similar for arm-cranking and leg-pedaling, but after training this cost was reduced by ~10% for arm-cranking, in association with decreases in VCO_2_, V_E_, V_t_, and HR, with no such changes with leg-pedaling. The relative VO_2_ (i.e., per kg of limb lean mass) was 56% lower for the legs, while the blood level of lactate rose 96% more after arm-cranking. After training, the ventilatory response at 80 W was similar for both sets of limbs, but as a result of the lower VO_2_ for the arms, their V_E_/VO_2_ and V_E_/VCO_2_ were greater. Thus, with the same whole-body VO_2_, the arms elicited a greater ventilatory response than the legs (Table 2).

#### Gross and delta efficiency

Prior to the training intervention, gross efficiency was higher for the legs than arms, but this variable was improved by training involving arm-cranking (~9%, *P* < 0.05), but not leg-pedaling (*P* = 0.25). Delta efficiency was 42% greater for the leg than arm exercise and not significantly altered by training (*P* = 0.43) (Table 2).

#### The time-trial

Following training the mean power output was 14.5 and 13.9% higher and the mean VO_2_ 11.4 and 7.9% higher for the arms and legs, respectively, with no significant change in blood levels of lactate (*P* = 1.00 and *P* = 0.92, respectively) (Table 3). During arm-cranking, the relative exercise intensity, HR, VO_2_, VCO_2_, V_E_, and V_t_ were all lower than during leg-pedaling, whereas the RER was higher during leg-pedaling. Oxidation of fat during the TT was minimal and remained so after training (Table 3). Despite the lower relative intensity during the TT with arm-cranking, fat oxidation was 70% greater during leg-pedaling (*P* = 0.017) (Table 3).

**Table 3:** **Effects of six sessions of sprint interval training on ergoespirometric variables and performance during a time-trial lasting for 5 min**.

	**Arms Pre-training**	**Arms Post-training**	**Legs Pre-training**	**Legs Post-training**	**Main training effect**	**Main extremity effect**	**Training × Extremity interaction**
Power (W)	118.2 ± 25.9	134.1 ± 29.8^a^	202.0 ± 42.2	225.8 ± 39.6^a^	0.000	0.000	0.740
HR (beats·min^−1^)	155.4 ± 10.3	156.1 ± 12.9	160.7 ± 11.4	164.4 ± 10.3	0.333	0.000	0.274
% VO_2_peak	67.7 ± 5.7	68.5 ± 8.4	77.3 ± 6.8	79.0 ± 5.4	0.490	0.000	0.639
VO_2_ (L·min^−1^)	2.13 ± 0.37	2.37 ± 0.47^a^	3.01 ± 0.51	3.25 ± 0.41^a^	0.002	0.000	0.960
VCO_2_ (L·min^−1^)	2.06 ± 0.39	2.33 ± 0.47^a^	2.87 ± 0.59	3.15 ± 0.42^a^	0.012	0.000	0.888
V*_E_* (L·min^−1^)	75.8 ± 18.7	81.3 ± 19.7	94.3 ± 27.8	101.4 ± 20.4	0.183	0.000	0.646
RER	0.95 ± 0.07	0.97 ± 0.05	0.92 ± 0.07	0.95 ± 0.04	0.227	0.034	0.696
RR (breaths·min^−1^)	37.4 ± 8.0	39.8 ± 8.8	34.3 ± 8.4	35.8 ± 7.0	0.238	0.026	0.416
V*_t_* (L)	2.05 ± 0.45	2.07 ± 0.48	2.71 ± 0.54	2.82 ± 0.41	0.275	0.000	0.153
V*_E_*/VO_2_	35.5 ± 5.6	34.3 ± 5.1	31.0 ± 6.1	31.1 ± 4.3	0.634	0.001	0.135
V*_E_*/VCO_2_	36.7 ± 4.3	34.9 ± 4.1	32.7 ± 5.2	32.1 ± 4.2	0.179	0.003	0.243
Blood Lactate (mM)	8.96 ± 1.76	8.88 ± 1.93	10.57 ± 2.37	10.80 ± 2.04	0.877	0.000	0.544
Fat oxidation (mg·min^−1^)	161.1 ± 146.8	110.6 ± 126.4	277.2 ± 224.2	185.5 ± 169.8	0.159	0.017	0.614

The values presented are means ± SD for the 5-min time-trial (n = 16); HR, Heart rate; VO_2_, mean oxygen uptake; VCO_2_, mean CO_2_ production; VE, mean pulmonary ventilation; RER, mean respiratory exchange ratio; RR, mean respiratory rate; Vt, mean tidal volume;

a *P < 0.05 compared to the corresponding Pre-training value*.

#### The wingate tests

PPO was elevated 10% for the arms and 5% for the legs by the intervention (Table 4). The MPO was 7% higher after training in the case of the arms and 5% for the legs. This elevation was the same in both Wingate tests for the arms (*P* = 0.759), but 3% greater in the second test with the legs (*P* < 0.05). The alterations in O_2_ demand paralleled those in MPO closely (Table 4). The PPO and MPO were significantly higher for the legs than arms in absolute terms, but greater for the arms in relation to lean mass.

**Table 4:** **Effects of six sessions of arm-cranking and leg pedaling sprint interval training on Wingate test performance and ergospirometric variables**.

**Variable**	**Wingate test**	**Arms**		**Legs**		**Main Training effect**	**Main W1 vs. W2**	**Interaction training × Wingate order**	**Arms W1 vs. W2**	**Legs W1 vs. W2**	**Main extremity effect**	**Interaction training × extremity**
		**Pre**	**Post**	**Pre**	**Post**							
PPO (W)	First	615.8 ± 89.8	659.3 ± 80.7	871.8 ± 84.9^b^	902.8 ± 84.4^c^	0.001	0.000	0.056	0.002	0.006	0.000	0.123
	Second	561.5 ± 80.8^d^	633.0 ± 85.4^a^	808.8 ± 119.8^b^	866.6 ± 106.9^c^							
PPO·lean mass^−1^ (W·kg^−1^)	First	74.8 ± 10.6	80.3 ± 6.7	42.1 ± 3.4^b^	43.5 ± 3.4^c^	0.001	0.000	0.046	0.002	0.006	0.000	0.091
	Second	68.0 ± 6.7^d^	77.1 ± 7.1^a^	39.0 ± 5.2^b^	41.7 ± 4.4^c^							
MPO (W)	First	431.6 ± 63.9	460.9 ± 61.1	607.6 ± 69.1^a^^,^ ^d^	626.7 ± 65.2^c^	0.000	0.000	0.403	0.000	0.000	0.000	0.332
	Second	381.9 ± 52.4^d^	413.4 ± 63.0^d^	533.4 ± 62.9^b^^,^ ^d^	567.0 ± 62.7^a^, ^c^^,^ ^d^							
MPO·lean mass^−1^ (W·kg^−1^)	First	52.4 ± 6.8	56.0 ± 3.5	29.2 ± 1.9^b^	30.1 ± 1.4^c^	0.000	0.000	0.324	0.000	0.000	0.000	0.265
	Second	46.2 ± 3.8 ^d^	50.3 ± 5.3^d^	25.6 ± 1.4^b^^,^ ^d^	27.2 ± 1.3^a^, ^c^^,^ ^d^							
O_2_ demand (L·min^−1^)	First	7.99 ± 1.20	8.14 ± 1.36	7.82 ± 1.07	8.41 ± 0.82	0.016	0.004	0.226	0.000	0.000	0.671	0.207
	Second	7.12 ± 0.86^d^	7.35 ± 1.33^d^	7.00 ± 0.99^d^	7.70 ± 0.77^d^							
O_2_ demand·kg BW^−1^ (ml·kg^−1^·min^−1^)	First	102.1 ± 14.0	104.0 ± 12.8	99.8 ± 10.9	108.1 ± 12.4	0.009	0.004	0.226	0.000	0.000	0.671	0.207
	Second	90.7 ± 6.6^d^	94.0 ± 13.7^d^	89.3 ± 10.5^d^	98.9 ± 10.7^d^							
O_2_demand·lean mass^−1^ (ml·min^−1^)	First	973.2 ± 157.7	988.0 ± 99.0	376.0 ± 35.2^b^	405.2 ± 38.8^c^	0.009	0.000	0.334	0.000	0.000	0.000	0.306
	Second	864.8 ± 96.1^d^	893.4 ± 116.9^d^	336.4 ± 32.2^b^^,^ ^d^	370.7 ± 33.3^c^^,^ ^d^							
VO_2_ (L)	First	0.94 ± 0.21	1.54 ± 0.30^a^	1.85 ± 0.23^b^	1.97 ± 0.17^c^	0.000	0.000	0.000	0.000	0.003	0.000	0.000
	Second	1.24 ± 0.18^d^	1.78 ± 0.21^a^	2.00 ± 0.20^b^	2.11 ± 0.20^a^, ^c^^,^ ^d^							
VO_2_·kg BW^−1^ (ml·kg^−1^·min^−1^)	First	12.0 ± 2.3	19.7 ± 3.4^a^	23.6 ± 2.7^b^	25.3 ± 1.8^c^	0.000	0.000	0.000	0.000	0.003	0.000	0.000
	Second	15.9 ± 2.0^d^	22.8 ± 2.0^a^^,^ ^d^	25.5 ± 2.1^b^	27.1 ± 1.8^a^, ^c^^,^ ^d^							
VO_2_·lean mass^−1^ (ml·kg^−1^·min^−1^)	First	113.7 ± 20.0	188.1 ± 34.8^a^	88.9 ± 7.7^b^	95.1 ± 6.7^c^	0.000	0.000	0.000	0.000	0.003	0.000	0.000
	Second	150.7 ± 17.1^d^	216.8 ± 18.3^a^^,^ ^d^	96.2 ± 5.9^b^	101.4 ± 4.0^a^, ^c^^,^ ^d^							
V*_E_* (L·min^−1^)	First	45.5 ± 11.9	59.5 ± 19.0	92.0 ± 28.9^b^	91.9 ± 20.3^c^	0.022	0.000	0.001	0.000	0.000	0.000	0.001
	Second	77.3 ± 12.6^d^	104.2 ± 17.4^a^^,^ ^d^	136.4 ± 24.5^b^^,^ ^d^	135.4 ± 21.7^c^^,^ ^d^							
RR (breaths·min^−1^)	First	40.7 ± 12.6	42.2 ± 18.1	64.8 ± 21.1^b^	57.7 ± 18.0	0.735	0.001	0.523	0.035	0.237	0.002	0.324
	Second	47.5 ± 10.5	49.0 ± 7.5	55.0 ± 12.1^b^	53.7 ± 9.9							
Tidal volume (L)	First	1.17 ± 0.29	1.50 ± 0.32^a^	1.47 ± 0.30^b^	1.64 ± 0.24	0.000	0.000	0.001	0.000	0.000	0.000	0.005
	Second	1.68 ± 0.33^d^	2.13 ± 0.22^a^^,^ ^d^	2.52 ± 0.37^b^^,^ ^d^	2.55 ± 0.36^c^^,^ ^d^							
O_2_ deficit (L)	First	3.05 ± 0.58	2.53 ± 0.64	2.07 ± 0.43^b^	2.23 ± 0.32	0.385	0.000	0.001	0.000	0.000	0.001	0.003
	Second	2.32 ± 0.38^d^	1.90 ± 0.51^a^^,^ ^d^	1.50 ± 0.39^b^^,^ ^d^	1.74 ± 0.27^d^							
O_2_ deficit·MPO^−1^ (mL·W^−1^)	First	7.10 ± 0.98	5.45 ± 0.96^a^	3.39 ± 0.54^b^	3.56 ± 0.44^c^	0.058	0.000	0.000	0.000	0.000	0.000	0.000
	Second	6.11 ± 0.92^d^	4.55 ± 0.75^a^^,^ ^d^	2.79 ± 0.53^b^^,^ ^d^	3.07 ± 0.44^c^^,^ ^d^							
O_2_ deficit·lean mass^−1^ (mL·Kg^−1^)	First	0.88 ± 0.19	0.67 ± 0.12^a^	0.16 ± 0.03^b^	0.17 ± 0.03^c^	0.057	0.000	0.000	0.000	0.000	0.000	0.001
	Second	0.75 ± 0.16^d^	0.56 ± 0.10^a^^,^ ^d^	0.13 ± 0.03^b^^,^ ^d^	0.15 ± 0.03^c^^,^ ^d^							

The values presented are means ± SD (n = 13, oxygen deficit could not be computed in all conditions in 3 subjects). W1, First Wingate test; W2, Second Wingate test; PPO, Peak power output; MPO, Mean power output; BW, Body weight; VO_2_, O_2_ consumption; Accumulated VO_2_, Total VO_2_ during the 30-s Wingate test; V_E_, Pulmonary ventilation; RR, Respiratory rate;

a P < 0.05 compared to the corresponding Pre value;

b P < 0.05 compared to the Pre value for the arms;

c P < 0.05 compared to the arms post;

d *P < 0.05 compared to W1*.

VO_2_ during the Wingate tests was on the average 23% greater after training –52% for the arms and 6% for the legs (*P* < 0.001). In the case of the arms, VO_2_ was higher during the first than the second Wingate test (69 vs. 44%, *P* < 0.05).

Training increased sprint V_E_ for arm-cranking only (+33%; *P* < 0.001, leg cycling *P* = 0.91), with no differences between the first and second sprints (*P* = 0.95), reflecting a more pronounced rise in tidal volume than during leg sprints (27 vs. 5%, *P* < 0.001), with no alteration in RR (*P* = 0.735). The O_2_ deficit remained unchanged during leg cycling, but was reduced by 17% during arm cycling, in both Wingate tests and even when normalized to the lean mass. Relative to MPO and lean mass, the O_2_ deficit was 4–5 fold greater for arm-cranking than leg-pedaling (Table 4). Blood levels of lactate during the first 3.5 min after W1 and W2 were unchanged by training (arms pre- and post-training: 8.49 ± 1.37 and 8.65 ± 1.09, respectively (*P* = 0.55); and legs pre- and post-training: 9.78 ± 1.76 to 10.14 ± 1.29 mM, respectively, P = 0.29; interaction *P* = 0.51), with a significantly greater response in the case of the legs than arms (*P* < 0.001).

### Muscle morphology

Apart from a small increase in the proportion of MHC-I in the *vastus* (*P* = 0.049), the distribution of fiber types was the same pre- and post-training. Analysis of MHC composition and ATPase in m. *vastus lateralis* and m. *triceps brachii* (distal part of the lateral head) revealed almost equal amounts of type I and type II fibers in the leg muscles (Table 5), while the *triceps brachii* contained predominantly type IIa fibers (Table 5). The CSA of type II fibers (IIa + IIx) was 39% greater in the m. *triceps brachii* than *vastus lateralis* (*P* < 0.05) (Table 5). After training, the capillary density per mm^2^ was 9% higher (*P* < 0.001). The mean number of capillaries in the vicinity of each fiber was 12% higher after training (*P* = 0.002), as a consequence of the 15% greater capillary density in the *triceps brachii* (*P* = 0.04). The number of capillaries per mm^2^ was 27% higher in the *vastus lateralis* than in the *triceps brachii* of the arms.

**Table 5:** **Lean mass, muscle morphology, and muscle enzymes before and after 7 sessions of sprint training**.

**Variable**	**Arms**		**Legs**		**Main training effect**	**Main extremity effect**	**Training × Extremity interaction**
	**Pre**	**Post**	**Pre**	**Post**			
Lean mass (g)	8223 ± 1111	8194 ± 1032	20769 ± 2015	20824 ± 2057	0.810	0.000	0.407
**ATPase ANALYSIS (*****n*** = **6)**							
Type I (%)^b^	42.3 ± 9.3	31.0 ± 5.7	51.9 ± 3.2	47.7 ± 11.4	0.116	0.010	0.180
Type IIa (%)^b^	56.9 ± 9.9	68.2 ± 6.2	46.5 ± 2.6	50.4 ± 10.2	0.119	0.004	0.260
Type IIx (%)^b^	2.5 ± 1.8	1.5 ± 1.2	2.1 ± 2.6	10.6			
Type II (IIa+IIx) (%)	57.7 ± 9.3	69.0 ± 5.7	47.9 ± 3.4	52.2 ± 11.5	0.112	0.010	0.194
CSA I (μm^2^)^b^	5232 ± 914	5729 ± 1191	4941 ± 473	4781 ± 669	0.572	0.163	0.223
CSA-IIa (μm^2^)^b^	8234 ± 1978	8102 ± 2722	6030 ± 707	5684 ± 1226	0.585	0.016	0.669
CSA-IIx (μm^2^)^b^	5929 ± 54	5701 ± 601	5873 ± 766	4044			
CSA-II (IIa+IIx) (μm^2^)^b^	8150 ± 1929	7983 ± 2795	5870 ± 837	5471 ± 979	0.492	0.024	0.757
**MYOSIN HEAVY CHAIN COMPOSITION (*****n*** = **16)**							
MHC I	26.4 ± 9.0	24.7 ± 7.3	44.4 ± 10.7	48.9 ± 11.4^a^	0.452	0.000	0.037
MHC IIa	67.8 ± 14.4	71.8 ± 8.9	52.8 ± 9.8	48.8 ± 11.4	0.996	0.000	0.061
MHC IIx	5.8 ± 9.8	3.5 ± 5.8	2.9 ± 3.0	2.3 ± 2.5	0.274	0.164	0.524
**CAPILLARY DENSITY (*****n*** = **15)**							
Capillaries·mm^−2^^c^	451 ± 114	477 ± 144	557 ± 139	618 ± 171^a^	0.004	0.001	0.234
Capillaries·fiber^−1^^c^	2.94 ± 0.72	3.38 ± 1.12^a^	2.95 ± 0.83	3.23 ± 0.69	0.002	0.754	0.596
**ENZYMES AND GLYCOGEN (*****n*** = **14–15)**							
Glycogen (mmol·kg^−1^)	422.6 ± 76.7	455.0 ± 47.7	428.5 ± 95.3	433.5 ± 70.8	0.182	0.816	0.576
CS (μmol·g dw^−1^·min^−1^)	33.7 ± 17.0	42.1 ± 13.6	71.9 ± 18.7	86.4 ± 18.0^a^	0.005	0.000	0.295
HAD (μmol·g dw^−1^·min^−1^)	58.7 ± 16.8	49.2 ± 15.1	96.2 ± 19.1	112.0 ± 25.8^a^	0.206	0.000	0.006

CSA, cross-sectional area; MHC, myosin heavy chain; CS, citrate synthase; HAD, hydroxyacyl-CoA-dehydrogenase; dw, dry weight;

a P < 0.05 compared to Pre-training;

b n = 6;

c *n = 15*.

#### Muscle levels of CS, HAD, and glycogen

As documented in Table 5, after training the CS and HAD activities were 20 and 16% higher in the *vastus lateralis*, but unaltered in the *triceps brachii*. The pre-training levels of CS and HAD activity were significantly higher in the *vastus lateralis* than the *triceps brachii*. The resting level of muscle glycogen was unaltered by the SIT (*P* = 0.51 and 0.93 for the arms and legs, respectively) and was similar in the arms and legs prior to the intervention (*P* = 0.99, Table 5).

#### Variables associated with time-trial performance

When pre- and post-training data were combined, time-trial performance (as reflected in the mean power output normalized to the lean mass) was positively associated with VO_2_peak per kg lean mass (*r* = 0.63 and 0.73 for the arms and legs, respectively, *P* < 0.001), MPO per kg lean mass (*r* = 0.57 and 0.42, *P* < 0.05), gross efficiency (*r* = 0.41 and 0.35, *P* < 0.05), CS activity (*r* = 0.46 and 0.46, *P* = 0.01), the proportion of MCH-I in the legs (*r* = 0.49, *P* = 0.004), delta efficiency in the arms (*r* = 0.42, *P* = 0.21), HAD activity in the arms (*r* = 0.38, *P* = 0.037), and blood lactate concentration in the legs at 80 W (*r* = −0.38, *P* = 0.030). The number of capillaries per fiber in the *triceps brachii* was correlated with the blood level of lactate 2.5 min after the incremental arm-cranking (*r* = 0.57 *P* < 0.001), as well as with gross efficiency (*r* = 0.38, *P* = 0.037), and VO_2_ peak per kg lean mass (*r* = 0.40, *P* = 0.027). The mean VO_2_ consumed during the two Wingate tests was associated with the MPO (arm-cranking: *r* = 0.60, *P* = 0.001, *n* = 26; leg-pedaling: *r* = 0.84, *P* < 0.001, *n* = 26) and VO_2_peak (arm-cranking *r* = 0.64, *P* < 0.001, *n* = 26; leg-pedaling *r* = 0.87, *P* < 0.001, *n* = 26). The mean O_2_ deficit during the two Wingate tests (in L·kg lean mass^−1^) was negatively associated with the CS activity in the arms (*r* = −0.45, *P* = 0.026, *n* = 24), but not in the legs (*r* = −0.05, *P* = 0.83, *n* = 24). No relationship was observed between the changes in the maximal respiration rate of isolated mitochondria (per mg wet tissue) and the time-trial performance (in watts per kg lean mass) (data not shown).

#### Variables that predicted time-trial performance

Multiple regression analysis revealed that the variables that predict TT performance with arm-cranking and leg**-**pedaling differ and, moreover, are altered in different ways by training (Table 6). Before training, VO_2_peak was the best predictor of TT performance by both the arms and legs, explaining 62 and 55% of these performances, respectively. After training, these values increased to 83 and 84%.

**Table 6:** **Multiple regression models predicting time-trail performance during arm cranking and leg pedaling before and the after training intervention**.

**Dependent**			**Unstandardized Coefficent**		**Standardized Coefficent**					
**Variable**	**Model**	**Variables**	***B***	***SD***	**Beta**	***t***	**Sig**.	***R*^2^**	**SEE**	**EV**
**ARM CRANKING BEFORE TRAINING**										
**Time-trial (W)** ANOVA *P* < 0.001	1	(Constant)	−52.42	46.82		−1.1	0.292	0.62	18.3	1,2,5,7,8,10,11
		VO_2_peak	53.51	14.02	0.786	3.8	0.004			
	2	(Constant)	40.09	47.50		0.8	0.423	0.812	13.6	
		VO_2_peak	51.00	10.48	0.749	4.9	0.001			
		Lac INCR	−8.08	2.82	−0.442	−2.9	0.021			
	3	(Constant)	−45.47	47.65		−1.0	0.372	0.908	10.2	
		VO_2_peak	47.62	7.95	0.7	6.0	0.001			
		Lac INCR	−6.41	2.20	−0.35	−2.9	0.023			
		Delta Efficiency	478.82	177.48	0.328	2.7	0.031			
	4	(Constant)	−162.50	29.91		−5.4	0.002	0.985	4.5	
		VO_2_peak	47.50	3.50	0.698	13.6	0.000			
		Lac INCR	−4.97	1.00	−0.271	−4.9	0.003			
		Delta Efficiency	606.05	81.50	0.415	7.4	0.000			
		Gross Efficiency	763.55	139.13	0.295	5.5	0.002			
	5	(Constant)	−183.20	16.45		−11.1	0.000	0.996	2.3	
		VO_2_peak	40.75	2.46	0.599	16.6	0.000			
		Lac INCR	−2.80	0.74	−0.153	−3.8	0.013			
		Delta Efficiency	714.72	50.19	0.49	14.2	0.000			
		Gross Efficiency	925.12	82.76	0.357	11.2	0.000			
		MHC I	−0.52	0.13	−0.187	−4.1	0.009			
**ARM CRANKING AFTER TRAINING**										
**Time-trial (W)** ANOVA *P* < 0.001	1	(Constant)	−94.15	40.63		−2.3	0.054	0.827	16.0	1,2,4,5,6,7,8,9,11
		VO_2_peak	65.34	11.28	0.91	5.8	0.001			
	2	(Constant)	−193.80	39.41		−4.9	0.003	0.94	10.2	
		VO_2_peak	63.46	7.21	0.884	8.8	0.000			
		%VO_2_peak	1.17	0.35	0.336	3.4	0.015			
	3	(Constant)	−205.46	28.34		−7.2	0.001	0.975	7.2	
		VO_2_peak	79.02	7.83	1.1	10.1	0.000			
		%VO_2_peak	1.14	0.25	0.327	4.6	0.006			
		Mean Oxygen Deficit	−18.15	6.91	−0.285	−2.6	0.047			
**LEG PEDALING BEFORE TRAINING**										
**Time-trial (W)** ANOVA *P* < 0.001	1	(Constant)	−45.12	81.15		−0.6	0.593	0.55	33.6	1,2,3,4,5,6,7,8,9,10,11
		VO_2_peak	63.26	20.38	0.739	3.1	0.015			
**LEG PEDALING AFTER TRAINING**										
**Time-trial (W)** ANOVA *P* < 0.001	1	(Constant)	−88.332	49.447		−1.786	0.112	0.843	18.0	1,2,3,4,6,7,8,9,10,11
		VO_2_peak	76.932	11.733	0.918	6.557	0			
	2	(Constant)	−211.027	44.264		−4.767	0.002			
		VO_2_peak	89.916	7.948	1.073	11.313	0	0.949	11.0	
		HAD	0.609	0.161	0.36	3.795	0.007			

*Variables code: 1, Capillaries; 2, Citrate synthase activity in mmol·kg^−1^ dry weight·min^−1^; 3, Delta efficiency; 4, Gross efficiency; 5, Hydroxyacyl-CoA-dehydrogenase activity (HAD) in μmol·g^−1^ dry weight·min^−1^; 6, Post-incremental exercise: blood lactate concentration (mmol·L^−1^) 2.5 min after exhaustion (Lac INCR); 7, Blood lactate concentration (mmol·L^−1^) during exercise at 80 W; 8, Myosin heavy chain Type I (MHC I) (%); 9, Myosin heavy chain Type IIa (MHC II) (%); 10, Mean oxygen deficit (litres) for the two Wingate tests (Mean OD); 11, Mean accumulated VO_2_ (litres) during the two Wingate tests; EV, variables excluded from the model with the highest predictive capacity*.

Prior to training, arm TT performance (TTa) in watts was predicted by the following equation:

(1) TTa = 40.8 · VO_2_peak − 2.8 · Lac INCR + 714.7 · DE + 925.1· GE–0.5 · MHC-I–183.2 (*R*^2^ = 0.996, *P* < 0.001).

The corresponding equation after training was:

(2) TTa = 79.0 · VO_2_peak + 1.1 · %VO_2_peak − 18.2 · Mean OD–205.5 (*R*^2^ = 0.98, *P* < 0.001).

Prior to training, leg time-trial performance was predicted by the following equation:

(3) TT_*L*_ = 63.3 · VO_2_peak − 45.1 (*R*^2^ = 0.55, *P* = 0.015).

and after training by the equation:

(4) TT_*L*_ = 89.9 · VO_2_peak + 0.6 · HAD–211 (*R*^2^ = 0.95, *P* < 0.001).

where VO_2_peak is expressed in L·min^−1^, the concentration of lactate 2.5 min after the end of the incremental exercise (Lac INCR) in mmol·L^−1^, %VO_2_peak in percentage units, mean OD in L·min^−1^, and HAD activity in μmol·g^−1^ dry weight·min^−1^.

The results of these calculations are documented in Table 6.

## Discussion

The major finding in this comparison of the responses of the arms and legs to SIT training in the same subjects was that the primary adaptation by both sets of limbs involves elevation of aerobic energy production. This occurred despite the fact that in both the trained and untrained state, the arms, with their higher percentage of type II fibers and lower activities of aerobic enzymes, were found to rely more heavily on anaerobic capacity during sprint exercise. VO_2_peak was the main determinant of endurance performance by arm and leg muscles both in the untrained and trained state, despite the different proportional contributions of anaerobic energy production in these limbs.

The performance of both sets of limbs in the 5-min time-trial improved to a similar extent, but this improvement was achieved by slightly different mechanisms. The enhanced arm sprint-performance was explained by the improvement in aerobic energy production, as reflected in the more pronounced elevation in VO_2_peak and greater enhancement of VO_2_ during the Wingate test, concomitant with a lower O_2_ deficit. Furthermore, gross efficiency was enhanced in the arms only. In contrast, VO_2_peak was improved more modestly by leg sprint training, despite the fact that the activities of CS and HAD rose significantly only in the leg muscles. Our results also show that both sets of limbs can be trained successively with a 1-h interval of rest with no negative cross-over effect on the limbs trained last.

### Comparison of the arms and legs

Even though the absolute values for key indicators of sprint performance (e.g., PPO and MPO) are generally lower for the arms than legs (Lutoslawska et al., 2003; Bouhlel et al., 2007; Zagatto et al., 2008), the PPO, MPO and whole-body VO_2_ in Wingate tests are almost twice as high for the arms when normalized to lean mass (Washburn and Seals, 1984; Sawka, 1986). However, the greater VO_2_ per kg of arm lean mass is also due to the fact that the VO_2_ by the arms accounts for only 1/3 of the whole-body peak VO_2_ during arm-cranking (Calbet et al., 2015a), whereas the leg VO_2_ accounts for 2/3 or more (Calbet et al., 2004, 2015a,b). The latter, combined with the almost 50% lower lean mass of the arms compared to the legs, results in a greater VO_2_peak for the arms, when VO_2_ is measured at the pulmonary level.

Nevertheless, when the VO_2_ by the muscles of the trunk, which represents 1/3 of the pulmonary VO_2_, is discounted (i.e., by determining O_2_ delivery and extraction using a-v differences in combination with blood flow assessment by thermodilution), the peak VO_2_ values per kg of arm or leg muscle are similar (Calbet et al., 2015a). This is in contrast to the fact that *in vitro* assessment of maximal mitochondrial respiration in permeabilized muscle fibers reveals higher values for the legs (Boushel et al., 2011, 2014a, 2015), indicating a greater functional reserve in mitochondrial VO_2_ in the leg than arm muscles. Although the arms contain a higher proportion of type II fibers, this does not appear to impede or limit their capacity to increase their VO_2_peak in response to sprint-training. In fact, certain reports have shown that in humans type II fibers display an aerobic capacity and metabolic phenotype similar to those of high-oxidative type I fibers (Proctor et al., 1995).

The gross efficiency of the arms was significantly lower than that of the legs, probably due to the greater contribution of resting and trunk VO_2_ to the overall energy expenditure by the arms. This interpretation is also supported by the closer correlation between the VO_2_ consumed during the Wingate pedaling tests and leg MPO (*r* = 0.84) than during the arm-cranking Wingates and arm MPO (*r* = 0.60).

### The primary mechanism of adaptation to sprint interval training involves enhancement of aerobic energy production

In several cases, as few as six sessions of “all-out” sprint intervals by untrained individuals have been reported to improve performance by the legs (Burgomaster et al., 2006; Gibala et al., 2006; Little et al., 2010). The current SIT enhanced the peak VO_2_ for the legs by 6%, which is similar to the 5–8% range observed by others following 2 weeks of SIT (Bailey et al., 2009; Hazell et al., 2010; Astorino et al., 2012; Jacobs et al., 2013). Here, we document the first assessment of the response of arm muscles to SIT. In contrast to our hypothesis, SIT increased VO_2_peak even more in the arms than the legs. Surprisingly, the oxygen deficit incurred during the sprints was not increased in the legs and was actually reduced in the arms, further indicating that the primary mechanism of adaptation to SIT involves enhanced production of aerobic energy. This agrees with findings of elevated muscle oxidative capacity determined by magnetic resonance spectroscopy following a similar leg SIT (Larsen et al., 2013).

However, this does not necessarily mean that SIT does not improve the production of anaerobic energy, e.g., from glycolysis and phosphocreatine. Some findings indicate increases in the activities of the enzymes that regulate glycolysis, greater glycogen storage and enhanced buffering capacity following a 2-week program of SIT (Rodas et al., 2000; Gibala et al., 2006), as well as improvement of lactate transport after more prolonged high-intensity interval training (Juel et al., 2004). Since anaerobic capacity does not limit short (30-s) sprint performance (Calbet et al., 2003; Morales-Alamo et al., 2012), enhancing this capacity should not necessarily improve performance during the two repeated 30-s sprints tested here. Moreover, it is unlikely that the entire anaerobic capacity was utilized in either of the two consecutive Wingate tests before or after the SIT, since O_2_ deficits of only 2–3 liters were reached (Medbø and Tabata, 1993; Calbet et al., 1997b).

**Figure 3:**
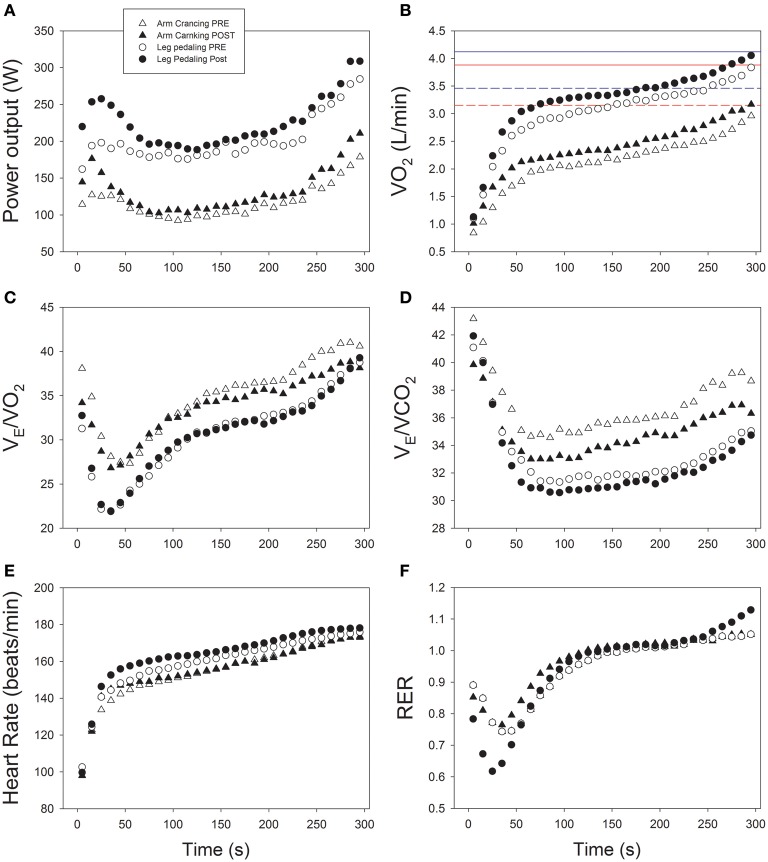
**Cardiorespiratory and ergometric responses to a 5-min time-trial before and after 6 sessions of sprint interval training during a 2-week period. (A)** Power output; **(B)** oxygen uptake (VO_2_); **(C)** Ventilatory equivalent for oxygen (V_E_/VO_2_); **(D)** Ventilatory equivalent for carbon dioxide (V_E_/VCO_2_); **(E)** heart rate; **(F)** respiratory exchange ratio (RER); *n* = 16.

Since each molecule of glucose metabolized aerobically yields 12–13 times as much ATP as catabolism of glucose to lactate, even a small enhancement in the capacity to utilize O_2_ will exert a pronounced impact on performance. Thus, the reduction in the O_2_ deficit of the arms during high-intensity exercise after SIT is the consequence of more rapid VO_2_ kinetics and a higher VO_2_peak (see Figure 3B), allowing more aerobic synthesis of ATP. This finding is supported by the observation that the blood lactate concentration after the Wingate tests was not increased by training. In agreement with our results, Bishop et al. (2009) reported a reduction in muscle buffering capacity and blood lactate accumulation after high-intensity exercise training, although with a quite different program of exercise than ours (5–8 bouts, 2 min at 100% VO_2_peak with 2-min recovery periods; 3 days/week for 5 weeks). Furthermore, the proportion of anaerobic ATP production from PCr will be high at the onset of exercise, especially during repeated sprints such as in SIT. Resynthesis of PCr is only possible under aerobic conditions (Harris et al., 1976) and will be enhanced by the increase in oxidative capacity caused by training, thereby contributing to the improved performance during the SIT and repeated Wingate tests.

SIT may have improved VO_2_peak by increasing oxygen delivery, but to date rebreathing procedures have detected no changes in cardiac output (Jacobs et al., 2013), while the response of blood flow in the arms and legs to SIT training has not yet been assessed in humans. Indicative of central cardiovascular adaptations, the submaximal (80 W) heart rate after training was 10% lower during arm-cranking, but not during leg-pedaling. This difference could reflect a greater improvement in peripheral O_2_ extraction during submaximal arm exercise, facilitated by the increased number of capillaries per fiber (Boushel et al., 2014a), which reduces the hyperemia required after SIT (Hellsten and Nyberg, 2015). Increasing the capillary density is not only advantageous for submaximal exercise, it also provides a functional reserve to increase peak blood flow without shortening mean transit time, that could otherwise occur when the peak blood flow increases without a concomitant enhancement of capillary density (Boushel et al., 2014a).

In addition, SIT might have reduced the perception of effort and, thereby, the central command during submaximal arm exercise, although there is no reason to believe that such a response would be more accentuated for the arms than legs, especially since the maximal exercise V_E_/VO_2_ and V_E_/VCO_2_ for both sets of extremities responded similarly to training. Nevertheless, a more pronounced afferent metaboreflex feedback might have been present during the TT with arm than leg exercise, as indicated by the greater relative hyperpnea (i.e., V_E_/VO_2_ and V_E_/VCO_2_) during the former, despite the slightly higher blood lactate concentration during the leg TT and the lower fraction of VO_2_peak during the arm TT (Dempsey et al., 2014; Torres-Peralta et al., 2016) (Figures 3C,D). Interestingly, the pacing strategy as well as the temporal V_E_/VO_2_ and V_E_/VCO_2_ responses were remarkably similar during the TT before and after training (Figure 3).

CS activity is considered a valid and robust marker of oxidative potential (Green et al., 1999; Larsen et al., 2012; Sloth et al., 2013). Following 2 weeks of SIT, CS activity in the legs has been reported to rise by 11–38% (Burgomaster et al., 2005, 2006), which is similar to the 20% elevation in the *vastus lateralis* and the non-significant 25% (*P* = 0.11) increase in the *triceps brachii* found in this study. The other key enzyme related to the aerobic potential of the muscles examined here was HAD, often used as indicator of the maximal capacity for fatty acid ß-oxidation (Gollnick et al., 1973). In agreement with previous studies (Burgomaster et al., 2006; Talanian et al., 2007), HAD activity in the leg muscles was elevated here. Considering the mean intensity and duration of our time-trial, oxidation of fat could not have been a limiting factor, so the increase in HAD activity is rather a proxy for elevated mitochondrial mass, as previously reported with SIT of 2 weeks duration (Jacobs et al., 2013), and which we confirmed in our subjects (Larsen et al., 2016). Consistent with this, fat oxidation during the 5-min time-trial was not altered in either the arms or legs by training. There were, however, no significant correlations between the improvement in the gross efficiency by the arms and the alterations in either CS or HAD (data not shown). Interestingly, despite a higher CS activity and increased VO_2_max, HAD activity in the arms was not changed, suggesting distinct metabolic responses by the two sets of limbs to training.

Unexpectedly, our subjects exhibited a reduced mitochondrial respiratory capacity due to high oxidative stress, resulting in inactivation of aconitase (Larsen et al., 2016). The fact that mitochondrial oxidative capacity does not limit VO_2_peak in humans (Boushel et al., 2011), and that peak VO_2_ can be maintained despite substantial reduction in mitochondrial capacity (Boushel et al., 2015; Larsen et al., 2016) further supports a more pronounced circulatory contribution to the present improvement in aerobic energy production in the arms. The number of capillaries per fiber in the arm muscles was higher after training, as previously reported for the *vastus lateralis* after a 6-week SIT (Cocks et al., 2013). This greater number of capillaries per fiber may have facilitated O_2_ diffusion and thereby enhanced O_2_ extraction and peak VO_2_ (Boushel et al., 2014a).

However, by measuring leg blood flow with thermodilution combined with arterial and venous femoral blood gases we have recently shown that there is a remarkable functional reserve in leg muscle diffusing capacity (DMO_2_) at VO_2_max. In subjects of similar characteristics as those included in the present investigation, DMO_2_ was 25.2±5.2 and 46.0±7.3 mL·min^−1^·mmHg^−1^ in normoxia and acute hypoxia (P_I_O_2_ = 74 mmHg, equivalent to 5300 m above sea level), respectively (Calbet et al., 2015b). In the same experiment DMO_2_ was even higher (51.5±9.7) during an isokinetic Wingate in hypoxia, despite the fact that before the sprint the subjects breathed a small amount of carbon monoxide, resulting in a 7.3% increase in carboxyhemoglobin (COHb). COHb left-shifted the oxygen dissociation curve, resulting in higher SaO_2_ during the sprint in hypoxia. The latter was combined with an almost similar leg blood flow, permitting greater maximal leg O_2_ delivery during sprint than during the incremental exercise to exhaustion in hypoxia and, hence, a greater leg VO_2_, despite a similarly low PaO_2_ (33.3 vs. 34.1 mmHg). This indicates that during leg sprint exercise VO_2_ is dependent on O_2_ delivery (Calbet et al., 2015b; Morales-Alamo et al., 2015). If SIT had not enhanced O_2_ delivery, then the only other mechanism that could explain an increase of VO_2_peak is enhancement of O_2_ extraction, which could have been facilitated by the increased capillarization and an increased mitochondrial affinity for O_2_, as suggested previously (Boushel et al., 2014b; Calbet et al., 2015b; Morales-Alamo et al., 2015).

### Why did the arms not adapt to SIT by increasing their production of anaerobic energy, despite their greater capacity to do so?

Blood lactate concentration and lactate release at the end of an incremental exercise to exhaustion are both elevated after prolonged high-intensity interval training (Juel et al., 2004). In the present investigation, the increase in capillary blood lactate concentration at the end of incremental arm-cranking to exhaustion is suggestive of an enhanced glycolytic capacity (Juel et al., 2004). This finding cannot be explained by slower lactate clearance, since no such increase was observed after incremental leg exercise following SIT, despite the greater muscle mass involved. Nevertheless, even if the anaerobic capacity was improved, this does not appear to have led to better performance during the Wingate test or the time-trials, possibly because anaerobic energy production does not limit performance during either all-out sprint exercise (Calbet et al., 2003; Morales-Alamo et al., 2012, 2015) or the 5-min time-trial employed here.

Moreover, our results indicate greater reliance on aerobic metabolism after training, implying less need for anaerobic generation of ATP during the 30-s arm-cranking sprint, as reflected in the lower oxygen deficit during the Wingate test after training. SIT enhanced arm performance without significant changes in the types or cross-sectional areas of muscle fibers or levels of aerobic enzymes.

### Factors limiting endurance performance

Obviously, to enhance performance, training must improve the factors that limit performance, which we examined here by multiple regression analysis. For both the arms and legs before and after training, the variable explaining most of the covariance in time-trial performance was the VO_2_peak, as reflected in the beta coefficients (Table 6). Prior to training, this coefficient was lower for the arms than the legs, indicating the presence of other predictive variables in the case of the arms. However, after training the beta coefficients for the VO_2_peak as a predictor of arm and leg TT performance both rose to the same value (1.1), indicating that in the trained state TT performance is even more dependent on VO_2_peak.

In the trained state, particularly in well-trained or elite athletes, endurance performance is considered to be determined by VO_2_max, the so-called “lactate threshold” and efficiency (Ingjer, 1991; Joyner and Coyle, 2008). The interaction between VO_2_max and the lactate threshold determines the highest fraction of the VO_2_max that can be sustained for any given period of time (Joyner and Coyle, 2008). This model of the factors that limit endurance performance is based primarily on measurements taken during cycling or running, i.e., leg exercise. Here, we report the first analysis of this nature on the same subjects exercising with either their arms or legs only.

Our findings indicate that the performance of untrained arms is limited by the VO_2_peak, the level of lactate 2.5 min after incremental arm exercise to exhaustion, delta and gross efficiencies, and the proportion of MHC-I (model 5 for arm-cranking before training; Table 6). As reflected in the beta coefficients, VO_2_peak, delta and gross efficiency are positive predictors, while the concentration of blood lactate post-incremental exercise and the percentage of type I MHC are negative predictors. In other words, prior to training greater reliance on anaerobic metabolism is negatively correlated to performance in a 5-min TT. The fact that MHC-I is a negative predictor implies that a greater fraction of MHC-II is advantageous for a 5-min TT with untrained arm muscles.

After training, the main predictors of TT performance change, so that the impact of VO_2_peak is even greater and the fraction of this value that can be sustained during the TT becomes a positive predictor, in accordance with current leg models (Joyner and Coyle, 2008). Interestingly, the oxygen deficit now becomes a negative predictor of arm TT performance, further emphasizing the conclusion that the major mechanisms of adaptation to SIT involve improvements in O_2_ delivery and/or utilization, i.e., the aerobic system. However, we cannot rule out the possibility that a reduced VO_2_ by trunk muscles following arm-cranking SIT played a major role in the improvement in the gross efficiency of the arms.

In the case of the legs, only VO_2_peak predicted TT pedaling performance before training, while after training, both VO_2_peak and HAD were positive predictors.

Thus, our present study demonstrates that the factors that limit endurance performance are somewhat different for the arms and legs and change in a somewhat different manner with training. When training enhances the capacity of a limiting factor, as for example, arm gross efficiency, it may no longer be limiting and may therefore disappear from the equation predicting performance.

### Limitations

Here, we demonstrate that the main mechanism of adaptation of both the legs and arms to SIT involves enhancement of the supply and utilization of aerobic energy. However, as cardiac output, limb blood flow and oxygen delivery were not measured, the relative roles played by central and peripheral mechanisms in the increase of VO_2_peak with training remains uncertain. Another limitation is related to the fact that the amount of active muscle is unknown, and the total mass of limb muscle was probably not recruited during maximal exercise. In the case of the arm exercise, the contribution by the muscles of the shoulders, torso, and legs is unknown. We presumed that this contribution was similar pre- and post-training, but this may be not the case. This same limitation applies to the contribution of muscles expanding proximally to the inguinal crease during leg exercise.

### Practical considerations

Although high-intensity and, in particular, sprint interval training is commonly employed by elite athletes to enhance performance, SIT is not typically recommended for others. For recreational athletes, as well as sedentary individuals and patients with chronic diseases, the recommendation has been endurance exercise of low-to-moderate intensity for 30–60 min daily or less frequent 20–60-min sessions of more vigorous exercise (Garber et al., 2011). However, this has changed recently and HIT is now being considered for such individuals as well (Wahl et al., 2010). Since the metabolic and health benefits of training are dependent on the amount of muscle employed, training both the upper- and lower-body may be beneficial, especially for untrained individuals and those with relatively little muscle mass in their lower-body.

Stationary cycling, which is non-weight-bearing and involves minimal eccentric contraction of the leg muscles, appears to minimize the risk of injury and discomfort. As shown here, even when training with the legs is not possible, training with the arms can improve aerobic capacity considerably within a short period of time. Thus, HIT may be considered for non-athlete populations after an adequate accommodation period. The duration of the present training intervention (six sessions in 2 weeks) was relatively short and it remains to be determined whether even more beneficial adaptations, especially in the arms, occur after a longer period of training.

Also of practical interest, our investigation demonstrates that training the arms before leg SIT training or vice-versa with 1 h of rest between the two sessions exerts no negative impact on adaptation by the limbs trained last. In moderately trained individuals, arm-cranking evokes more cardiovascular strain than leg-pedaling (Calbet et al., 2015a). It remains to be determined to what extent central cardiovascular adaptations elicited by arm training can be transferred to leg-pedaling and vice versa.

## Conclusions

The current investigation indicates that the main mechanism of adaptation of both the legs and arms to SIT involves enhancement of the supply and utilization of aerobic energy. Thus, when the same training program is applied to both sets of limbs, performance is enhanced in a similar manner, but through different mechanisms of local adaptation. Even though the arm muscles exhibit greater anaerobic potential, their adaptation to six sessions of SIT relies even more heavily on the improvement of VO_2_peak than in the case of the legs. The peak pulmonary VO_2_ and peak and mean power outputs (as assessed by Wingate tests) of both the arms and legs, as well as the mean power output during a 5-min time-trial were all enhanced after only six sessions of sprint interval training. Even though the arms contain a larger proportion of fast muscle fibers and exhibit lower mitochondrial VO_2_max *in vitro* and greater reliance on anaerobic energy production during sprint exercise, they are able to enhance their VO_2_peak relatively more than the legs in response to sprint training. We have also shown that the role played by VO_2_peak as a limiting factor for endurance performance becomes even more prominent after training. It remains to be determined whether an arm-cranking SIT can enhance VO_2_peak during subsequent leg exercise.

## Author contributions

All authors listed have made substantial, direct and intellectual contribution to this work, and approved it for publication.

## Funding

DM was a fellow of the Dr. Manuel Morales Foundation. No funding from the National Institutes of Health, the Welcome Trust, or the Howard Hughes Medical Institute was received for this work, which was financed by the institutions involved and by Ministerio de Economía y Competitividad (DEP2015-71171-R and DEP2009-11638 and FEDER).

### Conflict of interest statement

The authors declare that the research was conducted in the absence of any commercial or financial relationships that could be construed as a potential conflict of interest.

## Abbreviations

BW: body weight
COHb: carboxyhemoglobin
CS: citrate synthase
CSA: cross-sectional area
DMO_2_: leg muscle diffusing capacity
dw: dry weight
FI: fatigue index
HAD: 3-hydroxyacyl-CoA dehydrogenase
HIT: high-intensity training
HR: heart rate
OD: oxygen deficit
LM: lean mass
MHC: myosin heavy chains
MPO: mean power output during the WAnT
PPO: peak power output during the WAnT
RER: respiratory exchange ratio
RPE: ratings of perceived exertion
RR: respiratory rate
SDS: sodium dodecyl sulfate
SIT: sprint-interval training
T lim: duration of the incremental exercise test
TT: time trial
VCO_2_: carbon dioxide production
V_*E*_: pulmonary ventilation
VO_2_: oxygen consumption
VO_2_peak: maximal oxygen uptake
Vt: tidal volume
WAnT: Wingate anaerobic tests
Wmax: peak power output during the incremental exercise test.
